# Charting Health Challenges for Digital Preventive Interventions Among Adult Survivors of Childhood Acute Lymphoblastic Leukemia: National Long-Term Follow-Up Survey of Self-Rated Health Outcomes

**DOI:** 10.2196/54819

**Published:** 2024-08-12

**Authors:** Jens M Nygren, Katarina Aili, Susann Arvidsson, Maria Olsson, Marianne Jarfelt

**Affiliations:** 1 School of Health and Welfare Halmstad University Halmstad Sweden; 2 Department of Oncology Institute of Clinical Sciences, Sahlgrenska Academy University of Gothenburg Gothenburg Sweden; 3 Sahlgrenska University Hospital Gothenburg Sweden

**Keywords:** digital preventive interventions, long-term follow-up, self-rated health outcomes, adult survivors, childhood acute lymphoblastic leukemia

## Abstract

**Background:**

Acute lymphoblastic leukemia (ALL) is the most common malignancy in childhood, but the prognosis has remarkably improved over the last 50 years in high-income countries, and thus, there is a focus on long-term health outcomes following survival and how to best provide health care support to adult long-term survivors of childhood ALL to prevent and handle potential health problems. Digital health interventions are promising to deliver feasible health promotion and prevention programs. This is particularly relevant for ensuring long-term follow-up in cases where continuous contact with oncology care may be disrupted. Moreover, these interventions are beneficial in reaching geographically dispersed target groups and overcoming the time constraints of everyday life that often hinder participation in such programs.

**Objective:**

This study aimed to fill the gaps in existing research on adult long-term survivors of childhood ALL and provide formative data that can inform the development of formalized follow-up services designed to meet the needs of these survivors in ways that align with their preferences for digital health interventions.

**Methods:**

In this cross-sectional national study, adult survivors (aged ≥18 years) of childhood ALL for over 10 years after diagnosis were compared to their siblings in terms of mental and physical health-related factors, including sleep, stress, anxiety, and depression (Depression Anxiety and Stress Scale 21 [DASS-21]); several dimensions of fatigue (Multidimensional Fatigue Inventory 20 [MFI-20]); work ability (Work Ability Index); chronic pain; and prevalences of diabetes, cardiovascular disease, headache or migraine, and rheumatic disease.

**Results:**

Overall, 426 of 855 eligible ALL survivors responded (mean age 30.9, SD 7.7 years), and they participated at an average of 24 (SD 6.9) years after ALL diagnosis. Siblings (n=135; mean age 31.5, SD 7.7 years) acted as controls. Sleep quality, sleep quantity, and mean work ability scores were significantly lower, and physical fatigue, reduced motivation, and reduced activity scores were higher in ALL survivors than in siblings. There were no significant differences between the groups in terms of BMI and prevalence of chronic pain, depression, anxiety, or stress. Physical and psychological complications were more frequent among adult ALL survivors who had received hematopoietic stem cell transplantation (HSCT) than among those who had not received HSCT.

**Conclusions:**

Our nationwide cross-sectional study addressed the scarcity of knowledge regarding the self-reported health outcomes of adult long-term survivors of childhood ALL. We highlighted significant disparities within this population and emphasized the potential of comprehensive digital interventions that target vitality, sleep quality, fatigue, and psychosocial well-being to enhance well-being and bolster the capacity for managing chronic health conditions in this target group. Such an intervention would align with the needs of this target group, which is a prerequisite for successfully incorporating technology into the daily lives of survivors of childhood ALL.

## Introduction

Acute lymphoblastic leukemia (ALL) is the most common malignancy in childhood and accounts for approximately 30% of all childhood malignancies [1,2]. The prognosis has improved over the last 50 years in high-income countries, from a 5-year overall survival of approximately 5% in the 1970s to over 90% today [3-7]. Cranial irradiation improved the prognosis during the 1970s to 1980s but resulted in neurocognitive and endocrine complications, together with secondary malignancies [8,9]. Therefore, cranial irradiation has been replaced by high-dose methotrexate in most protocols, and for patients in whom cranial irradiation is indicated, the radiation dose has been reduced. However, neurocognitive complications are still present in this group [10]. In addition, for patients in need of hematopoietic stem cell transplantation (HSCT), the use of total body irradiation in conditioning regimens has so far been superior to other regimens, unfortunately with a high risk of several late complications [11-13]. Despite the continuous improvement in treatment regimens, the burden of chronic health conditions and the rate of hospitalization remain challenges in adult survivors of childhood ALL [14-16].

While previous studies have demonstrated a higher prevalence of chronic health conditions and a greater risk of poor health-related quality of life (HRQoL) among childhood ALL survivors [17], there is a relative scarcity of reports regarding other self-rated symptoms, which are essential for the overall health of adult long-term survivors of childhood ALL. These symptoms include psychological issues, fatigue, sleep disturbances, musculoskeletal pain, and self-rated work ability [18]. These outcomes may not have an immediate connection to the initial diagnosis of a chronic disease and are, in fact, commonly experienced by the general population. However, in the context of lifelong exposure owing to survivorship, these symptoms can accumulate and exert substantial impacts on both health and quality of life.

Identifying deviations and variations in these outcomes is of paramount importance. This is not solely to recognize individuals in need of immediate care and support but also to devise and offer interventions that can mitigate adverse health consequences and their impacts on daily life [19]. In Sweden, long-term follow-up guidelines for childhood cancer survivors have been present since 2016 [20]. This initiated the establishment of long-term follow-up clinics for adult childhood cancer survivors at 6 university hospitals. These clinics offer lifelong follow-up care for irradiated survivors, those who have received HSCT, and others with a high risk of late complications. However, for childhood ALL survivors treated solely with chemotherapy, the recommended practice is a single visit around the age of 25 years, with no further follow-up into adulthood. This aligns with guidelines indicating that limited doses of chemotherapy alone do not necessitate regular screening.

Traditional survivorship care has primarily focused on clinical follow-up and the long-term effects of therapy. However, there is a growing recognition of the importance of addressing broader aspects of survivorship, including quality of life, psychosocial well-being, and self-management of health. Participating in continuous health promotion and prevention programs could potentially be challenging for adult long-term survivors of childhood ALL, but digital approaches for such provisions have gained significant attention [19,21,22].

The use of digital services in long-term follow-up resonates with the preferences of long-term childhood cancer survivors. This demographic is accustomed to incorporating technology into their daily lives and often relies on it for seeking health-related information and support [21,22]. The advantages of digital services are numerous. They can be accessed at any time and from any location, making it easier for survivors to engage with health care resources without the constraints of geographic proximity or scheduling conflicts, and this is particularly beneficial for those who may have mobility issues or live in rural areas. They can provide personalized health information and support based on individual health data and preferences and therefore increase the likelihood of engaging users and helping them meet their specific needs. Moreover, they can facilitate continuous monitoring of health status and provide real-time feedback, supporting effective management of health issues and prompting timely interventions. The establishment of suitable and value-creating digital services for long-term follow-up opens up the possibility of integrating additional services beyond the follow-up of health issues, such as connecting survivors with peer support networks and fostering a sense of community and shared experience [23,24].

With existing knowledge on this subject being limited, the primary objective of this nationwide cross-sectional study was to investigate the prevalence of self-reported health and quality of life outcomes among adult survivors of childhood ALL who were diagnosed between 1985 and 2007. Additionally, this study sought to make comparisons between these outcomes in adult survivors and their siblings, who served as a control group with similar upbringing and living conditions, as well as between survivors who received HSCT and those who did not.

By focusing on these specific objectives, our study aims to fill the gaps in existing research on this specific target group of long-term survivors and provide formative data that can inform the development of formalized follow-up services that are designed to meet the needs of ALL survivors in ways that align with their preferences for digital health interventions.

## Methods

### Participants

In this cross-sectional population-based study, participants were recruited from the Swedish Childhood Cancer Registry, which is a national registry with a high coverage for childhood ALL since 1981 [25]. All individuals who were diagnosed with ALL between 1985 and 2007 at 0 to 18 years of age, were 18 years or older at the time of the study, had a period of over 10 years after diagnosis, and were able to self-report responses to the questionnaire, were eligible for participation.

### Recruitment

Contact details of the identified individuals were retrieved from the Swedish population register (SPAR, Statistics Sweden). Eligible participants were approached through letters including information about the study and a web link to the digital questionnaire (Multimedia Appendix 1) and a consent form. Overall, a maximum of 3 reminders were sent out, of which the third reminder included a paper version of the questionnaire. At the end of the questionnaire, participants had the option to suggest up to 2 of their siblings (age ≥18 years) to participate in the study by leaving contact details. The questionnaire and the procedure of reminders were the same for the siblings (Multimedia Appendix 2). All materials were presented in Swedish, and hence, only those who could read Swedish could participate in the study. Data were collected between January and August 2021 according to a previously described protocol [26]. Participants were not financially compensated for their contributions.

### Data Collection

Information on age, sex, year of ALL diagnosis, and type of treatment (HSCT) was collected directly from the Swedish Childhood Cancer Registry. Age was calculated in November 2021 when inclusion was terminated. Factors reflecting several outcomes on health and everyday life were assessed, including physical and mental health status and work ability. In addition, information on sociodemographic parameters was assessed, including marital status, having children, occupational status, and income.

BMI was calculated from self-reported height and weight (weight [in kg] divided by height [in meters]; kg/m^2^) and categorized as follows: (1) BMI of ≤18; (2) BMI of 19-24; (3) BMI of 25-29; and (4) BMI of ≥30.

Sleep quality was assessed by the item “How would you describe the quality of your sleep?” (referring to the last month). The response options were “good,” “sufficient,” and “poor.” Sleep quantity was assessed by the item “How many hours do you usually sleep per night?” The response options were <5 hours, 5-7 hours, 7-9 hours, and >9 hours.

Depression, anxiety, and stress were assessed by the Depression Anxiety and Stress Scale 21 (DASS-21), which is a short version of the DASS-42. Each of the 3 subscales is comprised of 7 items with responses reflecting 4 levels (0-3). To yield scores equivalent to scores in the DASS-42, the total score of each subscale is multiplied by 2, and thus, the scores range from 0 to 42. The subscores are categorized according to severity into normal, mild, moderate, severe, and extremely severe [27]. In this study, further categorization was adopted (“normal,” “mild/moderate,” and “severe/extremely severe”).

Chronic musculoskeletal pain was assessed by the key item “Have you experienced pain lasting more than 3 months during the last 12 months?” The question was followed by the presentation of a mannequin through which the location of the pain could be reported at 18 predefined bodily regions in the musculoskeletal system. The head and abdomen were not included [28,29]. Chronic widespread pain (CWP) was defined as included in the American College of Rheumatology 1990 criteria for fibromyalgia [30], according to which pain should be present in both the left and right sides of the body, above and below the waist, and in the axial skeleton. In addition, the pain should have lasted for 3 months or more to be considered chronic. If chronic pain was present (according to the key item), without fulfilling the criteria for CWP, the patients were classified as having chronic regional pain. Patients who had not experienced pain lasting more than 3 months during the last 12 months were categorized as having no chronic pain.

Work ability was assessed by the item “Current work ability compared with lifetime best” from the Work Ability Index (WAI). The item was scored from 0 (worst) to 10 (best) [31,32].

Fatigue was assessed by the Multidimensional Fatigue Inventory 20 (MFI-20) [33,34]. It consists of 20 items divided into 5 subscales: general fatigue; physical fatigue, reduced motivation, reduced activity, and mental fatigue. Each item is scored on a 5-point scale, and each subscale score ranges from 4 to 20, with a higher value indicating worse problems.

Comorbidity was assessed by items based on the Charlson Comorbidity Index [35], including the following: cardiovascular disease (CVD) (including items on ischemic heart disease, heart failure, bypass, and stroke), headache/migraine, diabetes, and rheumatoid disease (including rheumatoid arthritis, polymyalgia rheumatica, and systemic lupus erythematosus).

### Data Analysis

Analyses were performed to describe differences between the adult survivors of childhood ALL and their siblings regarding sociodemographic parameters and mental health and physical health parameters. Further, comparisons were performed between adult survivors of childhood ALL who had not received HSCT and those who had received HSCT, as well as between adult survivors of childhood ALL who had received HSCT and their siblings. For the comparison of means, 2-sided *t*-tests were performed. When comparing distributions by categorical variables, differences were evaluated by the chi-square test or Fisher exact test, as appropriate. All statistical analyses were performed using SPSS 26 (IBM Corp) without compensating for missing data.

### Ethical Considerations

This study was performed in accordance with the ethics approval obtained from the Swedish Ethics Review Authority (Dnr 2019-05181). Participation was based on voluntariness and informed consent. Participants could withdraw their participation whenever they wanted without having to provide a reason. All data were stored according to the Swedish University standards of research data storage and were handled with confidentiality. A key code was used to deidentify the primary data. It was kept separate and was only accessible to the first author. No remuneration was provided to the participants.

## Results

**Figure 1:**
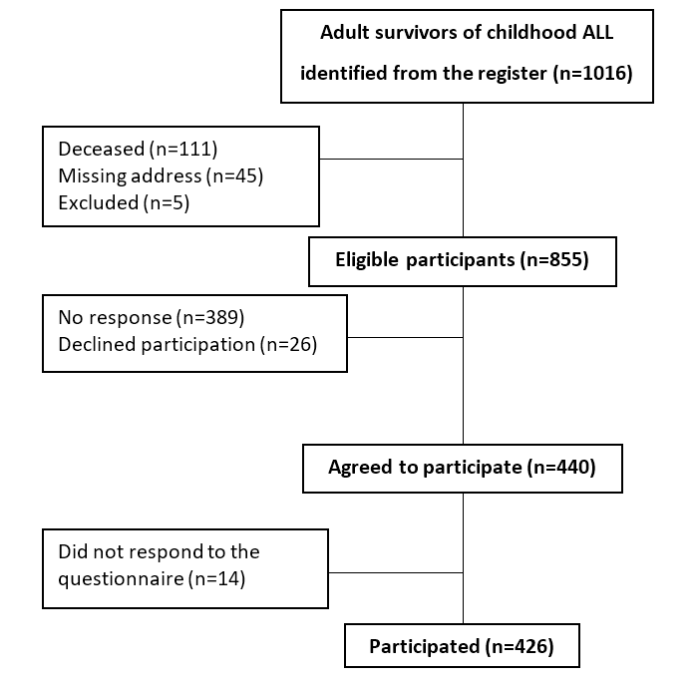
Flowchart describing the recruitment and response rate among the survivors of childhood ALL eligible for inclusion in the study. ALL: acute lymphoblastic leukemia.

Overall, 905 survivors of childhood ALL were identified, of which 45 had no address or a wrong address registered and could thus not be approached and 5 could not self-report responses to the questionnaire owing to Down syndrome functional disability (information provided by their caretakers when invited to the study). The remaining 855 survivors of childhood ALL were approached through letters, of whom 389 did not respond to the inquiry and 26 actively declined to participate. Of the 440 who agreed to participate, 14 did not respond to any of the items in the questionnaire, resulting in 426 (49.8%) of the 855 eligible survivors participating in the study (Figure 1). Of the 215 siblings identified by the participating survivors of childhood ALL, 8 could not be reached owing to wrong contact details and 135 (62.8%) responded to the same questionnaire as the survivors of childhood ALL.

Among the ALL survivors, between 13 and 36 years had passed since they had received their ALL diagnosis (mean 24, SD 6.9 years). There was no difference in the mean age between those who participated and those who did not participate (31.7 and 31.3 years, respectively; *P*=.48). Similarly, there was no difference in the mean age at diagnosis between participants and nonparticipants (6.3 and 6.7 years, respectively; *P*=.16). There was a higher proportion of women among those who participated in the study than among those who did not (208/426, 48.8% vs 172/429, 40.1%; *P*=.01). There were significantly more ALL survivors who had been treated with HSCT among those who participated than among those who did not participate (33/426, 7.7% vs 24/429, 5.6%; *P*<.001). However, information on HSCT was missing more frequently in the registry among those who did not participate than among those who participated (152/426, 35.7% vs 91/429, 21.2%; *P*<.001).

The adult survivors of childhood ALL had a similar mean age as the group of siblings (30.9 and 31.5 years, respectively). However, the maximum age was somewhat higher among siblings than among ALL survivors (range 18-54 years and 18-49 years, respectively), with the difference being significant (*P*=.04) but most likely not relevant. The female-to-male distribution was more among survivors of childhood ALL (209/426, 49.0% females) than among siblings (84/135, 62.2% females). A larger proportion of siblings than ALL survivors was married or cohabiting (90/135, 66.7% vs 231/426, 54.2%) or in a relationship (13/135, 9.6% vs 34/426, 8.0%), and it was more common for siblings than ALL survivors to have children (68/135, 50.4% vs 167/426, 39.3%). There were no significant differences between the groups regarding occupational status; however, monthly income was significantly lower among ALL survivors than among siblings (*P*<.001) (Table 1).

There were no significant differences between the groups in BMI, mental health (depression, anxiety, and stress), and the prevalence of chronic musculoskeletal pain or comorbidities (CVD, diabetes, rheumatic disease, or headache). However, although not statistically significant, a larger proportion of ALL survivors than siblings had CVD (31/407, 7.6% vs 4/125, 3.2%) and rheumatic disease (17/405, 4.0% vs 1/125, 0.8%). Childhood ALL survivors had worse sleep quality (*P*=.009) and sleep quantity (*P*=.046), had inferior work ability (*P*=.01), scored worse on physical fatigue (*P*<.001), and reported reduced motivation (*P*<.001) and reduced activity (*P*<.001) compared with the findings in siblings (Table 2).

Among the childhood ALL survivors, 33 had received HSCT, while 303 had not received HSCT. There was no difference in mean age between those who had received HSCT and those who had not. The number of chronic pain sites was significantly higher among childhood ALL survivors who had received HSCT than among survivors who had not received HSCT (*P*=.02). Moreover, there was a difference in the prevalence of CWP; however, the difference was not statistically significant (*P*=.051). The prevalence of diabetes was higher and work ability was worse among survivors who had received HSCT than among those who had not received HSCT (*P*=.007 and *P*=.01, respectively). However, no differences between the groups were seen in terms of BMI, sleep, stress, anxiety, depression, fatigue, and the prevalence of CVD, headache, or rheumatic disease (Table 3).

Finally, in a comparison between childhood ALL survivors who had received HSCT and the siblings, the former had significantly impaired sleep quality (*P*=.009) and sleep quantity (*P*=.03) and were more likely to report severe depression (*P*=.01), chronic pain (*P*=.01), a higher number of pain sites (*P*=.007), worse work ability (*P*=.003), more physical fatigue (*P*<.001), reduced motivation (*P*<.001), and reduced activity (*P*<.001). The prevalence of diabetes was higher among ALL survivors who had received HSCT than among the siblings (*P*=.006), and the prevalence of CVD was also higher among ALL survivors who had received HSCT, but the difference was not statistically significant (*P*=.055) (Table 4).

**Table 1:** Characteristics of adult survivors of childhood acute lymphoblastic leukemia and their siblings.

Characteristic		Adult survivors of childhood ALL^a^ (n=426), n (%)	Siblings (n=135), n (%)	*P* value^b^	
**Age group (years)**					.08
	18-24	114 (26.8)	24 (17.8)		
	25-34	148 (34.7)	57 (42.2)		
	≥35	164 (38.5)	54 (40.0)		
**Sex**					.008
	Male	217 (51.0)	51 (37.8)		
	Female	209 (49.0)	84 (62.2)		
**Marital status**					.02
	Married/cohabiting	231 (54.2)	90 (66.7)		
	Single	159 (37.3)	31 (23.0)		
	In a relationship	34 (8.0)	13 (9.6)		
	Other	2 (0.5)	1 (0.7)		
**Children**					.02
	Yes	167 (39.2)	68 (50.4)		
	No	259 (60.8)	67 (49.6)		
**Occupational status**					.16
	Studying	76 (17.8)	22 (16.3)		
	Working	276 (64.8)	97 (71.8)		
	Unemployed	19 (4.5)	2 (1.5)		
	On parental leave	14 (3.2)	8 (6.0)		
	On sick leave	22 (5.2)	3 (2.2)		
	Voluntarily not working	2 (0.5)	1 (0.7)		
	Other	17 (4.0)	2 (1.5)		
**Monthly income^c^**					<.001
	<10,000	72 (16.9)	12 (8.9)		
	10,000-19,999	73 (17.1)	25 (18.5)		
	20,000-29,999	110 (25.8)	19 (14.1)		
	30,000-39,999	102 (24.0)	44 (32.6)		
	40,000-49,999	47 (11.0)	18 (13.3)		
	50,000-59,999	14 (3.3)	7 (5.2)		
	≥60,000	8 (1.9)	10 (7.4)		

^a^ALL: acute lymphoblastic leukemia.

^b^Difference in distribution between the groups by the chi-square test.

^c^Income is presented in Swedish krona (SEK); 10,000 SEK is equivalent to approximately 925 USD.

**Table 2:** Health-related factors in adult survivors of childhood acute lymphoblastic leukemia (n=426) and their siblings (n=135).

Characteristic		Adult survivors of childhood ALL^a^	Siblings	*P* value^b^	
**BMI, n/N (%)**					.33^c^
	≤18	17/415 (4.1)	6/133 (4.5)		
	19-24	212/415 (51.1)	73/133 (54.9)		
	25-29	130/415 (31.3)	44/133 (33.1)		
	≥30	56/415 (13.5)	10/133 (7.5)		
**Sleep quality, n/N (%)**					.009^c^
	Good	171/425 (40.3)	66/135 (48.9)		
	Sufficient	160/425 (37.6)	55/135 (40.7)		
	Poor	94/425 (22.1)	14/135 (10.4)		
**Sleep quantity, n/N (%)**					.046^c^
	<5 hours	23/425 (5.4)	6/135 (4.4)		
	5-7 hours	185/425 (43.5)	50/135 (37.0)		
	7-9 hours	203/425 (47.8)	79/135 (58.6)		
	>9 hours	14/425 (3.3)	0/135 (0.0)		
**Stress, n/N (%)**					.49^c^
	Normal	301/396 (76.0)	99/124 (79.9)		
	Mild/moderate	68/396 (17.2)	20/124 (16.1)		
	Severe/very severe	27/396 (6.8)	5/124 (4.0)		
**Anxiety, n/N (%)**					.42^c^
	Normal	295/407 (72.5)	98/125 (78.4)		
	Mild/moderate	64/407 (15.7)	15/125 (12.0)		
	Severe/very severe	48/407 (11.8)	12/125 (9.6)		
**Depression, n/N (%)**					.13^c^
	Normal	265/408 (65.0)	92/125 (73.6)		
	Mild/moderate	100/408 (24.5)	26/125 (20.8)		
	Severe/very severe	43/408 (10.5)	7/125 (5.6)		
**Chronic pain, n/N (%)**					.32^c^
	NCP^d^	280/407 (68.8)	93/125 (74.4)		
	CRP^e^	97/407 (23.8)	27/125 (21.6)		
	CWP^f^	30/407 (7.4)	5/125 (4.0)		
Number of pain sites (N=407 for survivors; N=125 for siblings), mean (SD)		1.43 (2.81)	0.97 (2.47)	.08^g^	
Work ability^h^ (N=424 for survivors; N=135 for siblings), mean (SD)		8.17 (2.46)	8.66 (1.86)	.01^g^	
General fatigue^i^ (N=407 for survivors; N=125 for siblings), mean (SD)		11.17 (2.13)	11.49 (4.12)	.41^g^	
Physical fatigue^i^ (N=406 for survivors; N=125 for siblings), mean (SD)		12.09 (2.12)	9.46 (4.42)	<.001^g^	
Reduced motivation^i^ (N=407 for survivors; N=125 for siblings), mean (SD)		12.07 (2.12)	8.25 (3.30)	<.001^g^	
Reduced activity^i^ (N=407 for survivors; N=125 for siblings), mean (SD)		12.85 (2.45)	9.95 (3.92)	<.001^g^	
Mental fatigue^i^ (N=407 for survivors; N=125 for siblings), mean (SD)		10.46 (2.23)	10.57 (3.43)	.75^g^	
Diabetes (yes), n/N (%)		10/406 (2.5)	1/125 (0.8)	.25^g^	
CVD^j^ (yes), n/N (%)		31/407 (7.6)	4/125 (3.2)	.08^g^	
Headache/migraine (yes), n/N (%)		185/407 (45.5)	61/125 (48.8)	.51^g^	
Rheumatic disease (yes), n/N (%)		17/405 (4.2)	1/125 (0.8)	.07^g^	

^a^ALL: acute lymphoblastic leukemia.

^b^Group differences by the *t-*test or chi-square test as appropriate.

^c^Chi-square test.

^d^NCP: no chronic pain.

^e^CRP: chronic regional pain.

^f^CWP: chronic widespread pain.

^g^*t-*test.

^h^From the Work Ability Index (score 0-10).

^i^From the Multidimensional Fatigue Inventory (score 4-20).

^j^CVD: cardiovascular disease.

**Table 3:** Health-related factors in adult survivors of childhood acute lymphoblastic leukemia who had not received hematopoietic stem cell transplantation (n=303) and those who had received hematopoietic stem cell transplantation (n=33).

Characteristic			Survivors of childhood ALL^a^ who had not received HSCT^b^		Survivors of childhood ALL who had received HSCT		*P* value^c^	
Age (N=303 for no HSCT; N=33 for HSCT), mean (SD)			30.5 (7.6)		31.9 (6.4)		.31	
**BMI, n/N (%)**								.52^d^
	≤18	11/295 (3.7)		1/31 (3.2)				
	19-24	147/295 (49.9)		19/31 (61.3)				
	25-29	93/295 (31.5)		9/31 (29.0)				
	≥30	44/295 (14.9)		2/31 (6.5)				
**Sleep quality, n/N (%)**								.34
	Good	125/302 (41.4)		10/33 (30.3)				
	Sufficient	114/302 (37.7)		13/33 (39.4)				
	Poor	63/302 (20.9)		10/33 (30.3)				
**Sleep quantity, n/N (%)**								.42^d^
	<5 hours	16/302 (5.3)		3/33 (9.1)				
	5-7 hours	128/302 (42.4)		14/33 (42.4)				
	7-9 hours	149/302 (49.3)		14/33 (42.4)				
	>9 hours	9/302 (3.0)		2/33 (6.1)				
**Stress, n/N (%)**								.70 ^d^
	Normal	217/283 (76.7)		22/30 (73.3)				
	Mild/moderate	46/283 (16.2)		5/30 (16.7)				
	Severe/very severe	20/283 (7.1)		3/30 (10.0)				
**Anxiety, n/N (%)**								.61^d^
	Normal	210/292 (71.9)		22/32 (68.7)				
	Mild/moderate	45/292 (15.4)		4/32 (12.5)				
	Severe/very severe	37/292 (12.7)		6/32 (18.8)				
**Depression, n/N (%)**								.18
	Normal	189/292 (64.7)		18/32 (56.2)				
	Mild/moderate	72/292 (24.7)		7/32 (21.9)				
	Severe/very severe	31/292 (10.6)		7/32 (21.9)				
**Chronic pain, n/N (%)**								.051
	NCP^e^	199/291 (68.4)		16/32 (50.0)				
	CRP^f^	74/291 (25.4)		11/32 (34.4)				
	CWP^g^	18/291 (6.2)		5/32 (15.6)				
Number of pain sites (N=291 for no HSCT; N=32 for HSCT), mean (SD)			1.32 (2.57)		3.12 (4.09)		.02	
Work ability^h^ (N=301 for no HSCT; N=33 for HSCT), mean (SD)			8.31 (2.27)		6.73 (3.42)		.01	
General fatigue^i^ (N=291 for no HSCT; N=32 for HSCT), mean (SD)			11.14 (2.10)		11.09 (2.35)		.91	
Physical fatigue^i^ (N=290 for no HSCT; N=32 for HSCT), mean (SD)			12.02 (2.09)		12.22 (2.27)		.61	
Reduced motivation^i^ (N=291 for no HSCT; N=32 for HSCT), mean (SD)			12.07 (2.72)		12.22 (2.27)		.78	
Reduced activity^i^ (N=291 for no HSCT; N=32 for HSCT), mean (SD)			12.86 (2.42)		13.16 (2.38)		.51	
Mental fatigue^i^ (N=291 for no HSCT; N=32 for HSCT), mean (SD)			10.40 (2.11)		10.19 (2.58)		.61	
Diabetes (yes), n/N (%)			5/291 (1.7)		4/32 (12.5)		.007^d^	
CVD^j^ (yes), n/N (%)			19/291 (6.5)		4/32 (12.5)		.27^d^	
Headache/migraine (yes), n/N (%)			132/291 (45.4)		15/32 (46.9)		.87	
Rheumatic disease (yes), n/N (%)			12/290 (4.1)		2/32 (6.3)		.64^d^	

^a^ALL: acute lymphoblastic leukemia.

^b^HSCT: hematopoietic stem cell transplantation.

^c^Group differences by the *t*-test or chi-square test as appropriate.

^d^Fisher exact test.

^e^NCP: no chronic pain.

^f^CRP: chronic regional pain.

^g^CWP: chronic widespread pain.

^h^From the Work Ability Index (score 0-10).

^i^From the Multidimensional Fatigue Inventory (score 4-20).

^j^CVD: cardiovascular disease.

**Table 4:** Health-related factors in adult survivors of childhood acute lymphoblastic leukemia who had received hematopoietic stem cell transplantation (n=33) and the siblings (n=135).

Characteristic			Survivors of childhood ALL^a^ who had received HSCT^b^		Siblings		*P* value^c^	
**BMI, n/N (%)**								.96^d^
	≤18	1/31 (3.2)		6/133 (4.5)				
	19-24	19/31 (61.3)		73/133 (54.9)				
	25-29	9/31 (29.0)		44/133 (33.1)				
	≥30	2/31 (6.5)		10/133 (7.5)				
**Sleep quality, n/N (%)**								.009
	Good	10/33 (30.3)		66/135 (48.9)				
	Sufficient	13/33 (39.4)		55/135 (40.7)				
	Poor	10/33 (30.3)		14/135 (10.4)				
**Sleep quantity, n/N (%)**								.03^d^
	<5 hours	3/33 (9.1)		6/135 (4.4)				
	5-7 hours	14/33 (42.4)		50/135 (37.1)				
	7-9 hours	14/33 (42.4)		79/135 (58.5)				
	>9 hours	2/33 (6.1)		0/135 (0.0)				
**Stress, n/N (%)**								.36^d^
	Normal	22/30 (73.3)		99/124 (79.9)				
	Mild/moderate	5/30 (16.7)		20/124 (16.1)				
	Severe/very severe	3/30 (10.0)		5/124 (4.0)				
**Anxiety, n/N (%)**								.32^d^
	Normal	22/32 (68.8)		98/125 (78.4)				
	Mild/moderate	4/32 (12.5)		15/125 (12.0)				
	Severe/very severe	6/32 (18.8)		12/125 (9.6)				
**Depression, n/N (%)**								.01
	Normal	18/32 (56.2)		92/125 (73.6)				
	Mild/moderate	7/32 (21.9)		26/125 (20.8)				
	Severe/very severe	7/32 (21.9)		7/125 (5.6)				
**Chronic pain, n/N (%)**								.01
	NCP^e^	16/32 (50.0)		93/125 (74.4)				
	CRP^f^	11/32 (34.4)		27/125 (21.6)				
	CWP^g^	5/32 (16.6)		5/125 (4.0)				
Number of pain sites (N=32 for survivors who received HSCT; N=125 for siblings), mean (SD)			3.12 (4.09)		0.97 (2.47)		.007	
Work ability^h^ (N=33 for survivors who received HSCT; N=135 for siblings), mean (SD)			6.73 (3.42)		8.66 (1.86)		.003	
General fatigue^i^ (N=32 for survivors who received HSCT; N=125 for siblings), mean (SD)			11.09 (2.35)		11.49 (4.12)		.48	
Physical fatigue^i^ (N=32 for survivors who received HSCT; N=125 for siblings), mean (SD)			12.22 (2.27)		9.46 (4.42)		<.001	
Reduced motivation^i^ (N=32 for survivors who received HSCT; N=125 for siblings), mean (SD)			12.22 (2.27)		8.25 (3.30)		<.001	
Reduced activity^i^ (N=32 for survivors who received HSCT; N=125 for siblings), mean (SD)			13.16 (2.38)		9.95 (3.92)		<.001	
Mental fatigue^i^ (N=32 for survivors who received HSCT; N=125 for siblings), mean (SD)			10.19 (2.58)		10.57 (3.43)		.25	
Diabetes (yes), n/N (%)			4/32 (12.5)		1/125 (0.8)		.006^d^	
CVD^j^ (yes), n/N (%)			4/32 (12.5)		4/125 (3.2)		.055^d^	
Headache/migraine (yes), n/N (%)			15/32 (46.9)		61/125 (48.8)		.85	
Rheumatic disease (yes), n/N (%)			2/32 (6.3)		1/125 (0.8)		.11^d^	

^a^ALL: acute lymphoblastic leukemia.

^b^HSCT: hematopoietic stem cell transplantation.

^c^Group differences by the *t*-test or chi-square test as appropriate.

^d^Fisher exact test.

^e^NCP: no chronic pain.

^f^CRP: chronic regional pain.

^g^CWP: chronic widespread pain.

^h^From the Work Ability Index (score 0-10).

^i^From the Multidimensional Fatigue Inventory (score 4-20).

^j^CVD: cardiovascular disease.

## Discussion

### Principal Findings

This study delves into the self-rated outcomes pertaining to physical health, psychosocial well-being, and socioeconomic factors in adult survivors of childhood ALL. Our primary goal was to illuminate the disparities between these survivors and a control group composed of their siblings, revealing marked differences across all 3 domains. Particularly striking were the distinctions in vitality, with adult survivors of childhood ALL experiencing a higher prevalence of sleep disturbances, diminished work ability, and increased physical fatigue compared to their siblings. Furthermore, the adult ALL survivors who underwent HSCT exhibited even greater occurrences of both physical and psychological complications. This study identifies potential focal points for preventive interventions and emphasizes that these interventions should not solely address specific chronic health conditions relevant to this target group or general issues related to the risk of diminished HRQoL among childhood ALL survivors. On the contrary, interventions should take a broader approach by focusing on enhancing everyday functionality, particularly in terms of helping individuals manage the difficulties associated with vitality, sleep disturbances, work ability, and physical fatigue. By doing so, we can significantly enhance their well-being, bolster their capacity to cope with chronic conditions, and hopefully reduce the likelihood of more severe health complications later in life.

### Health Outcomes of Adult Survivors of Childhood ALL

Adult survivors of childhood ALL reported poor sleep twice as frequently as their siblings, with both shorter and longer sleep durations being more common among ALL survivors. Notably, the group that had received HSCT showed no difference in sleep when compared to other ALL survivors, but they were 3 times more likely to report poor sleep and more likely to report both shorter and longer sleep durations when compared to their siblings. It is important to recognize that poor sleep, defined as less than 6 hours of sleep per night, is linked to increased mortality and adverse health outcomes, including CVD, diabetes, and obesity [36,37].

Prior studies have reported conflicting findings regarding fatigue in childhood cancer survivors and childhood ALL survivors [38-41]. Our study revealed that survivors of childhood ALL reported higher physical fatigue than their siblings but exhibited similar levels of general and mental fatigue. This elevated physical fatigue among adult survivors of childhood ALL may suggest physical effects resulting from treatment-related factors [42,43]. Our study revealed reduced sleep quantity among ALL survivors, which has previously been associated with impaired neurocognitive function when accompanied by daytime sleepiness [44]. Moreover, although we found no difference in mental fatigue, motivation was lower among adult survivors of childhood ALL, potentially indicating mental fatigue. Neurotoxic treatments used for ALL have been previously linked to neurocognitive deficits [43,45,46], which could indirectly affect motivation and work ability. Moreover, frailty, which has been shown to be associated with neurocognitive decline in adult childhood cancer survivors [47], may manifest as physical fatigue, influencing both motivation and work ability. Further research is necessary to investigate the potential associations between late physical and neurocognitive issues and lifestyle habits in this patient group.

While there was no significant difference in reported anxiety or depression overall, those who had received HSCT had a higher incidence of depression compared to their siblings. Additionally, stress levels did not differ between survivors and their siblings. Earlier studies involving adult childhood cancer survivors have yielded mixed results regarding depression and distress compared to the general population [48-50], and increased incidences of depression and anxiety have been reported in childhood ALL survivors during childhood and adolescence [51]. However, the results for adult survivors of childhood ALL are inconsistent [52,53].

In our study, adult survivors of childhood ALL did not significantly differ from their siblings in terms of chronic pain prevalence. However, there was a tendency toward a higher prevalence of rheumatic diseases among adult survivors of childhood ALL compared to their siblings and the general population. This aligns with previous findings of a higher incidence of autoimmune diseases in survivors of childhood ALL [54]. In the group that had received HSCT, there was a tendency toward increased pain and a significantly higher number of pain sites compared to those who had not received HSCT. The reported CWP was up to 4 times as common among adult survivors of childhood ALL who had received HSCT than among their siblings. In general population studies, the prevalence of CWP in adults was approximately 10%, increasing with age [28,55,56]. Given the relatively low mean age in our study, the expected prevalence of CWP should be lower than 10% but was as high as 16% among those who had received HSCT. Although ALL treatment, which always includes vinca alkaloids, can lead to chronic neuropathies and high doses of corticosteroids can induce osteonecrosis [57], these conditions are not typically associated with CWP. Our results are consistent with the findings of prior studies involving adult survivors of childhood cancer, where pain was reported to be relatively low but more frequently linked to chronic health conditions and previous radiation exposure, which could account for our findings among those treated with HSCT [57,58]. Additionally, graft-versus-host disease in HSCT survivors can lead to musculoskeletal deficits [59]. Furthermore, our study identified a significantly higher incidence of diabetes in adult survivors of childhood ALL treated with HSCT, which was consistent with the findings of previous research showing an increased incidence of diabetes in childhood ALL survivors, particularly when treatment exposes the pancreas to radiation, as is the case for survivors of childhood ALL treated with HSCT [60,61].

### Health Services Targeting Adult Survivors of Childhood ALL

Our study targeted a specific diagnosis group of long-term survivors of ALL. Since ALL is the most common childhood malignancy with a high survival rate, this is the largest group of childhood cancer survivors. The focus on ALL survivors alone allows for the presentation of data with a high level of specificity. This granularity is crucial for understanding the unique long-term effects and health care needs of this survivor group. Unlike many long-term follow-up studies that rely solely on clinical objective data, our research is based on self-reported data from survivors. This approach provides valuable insights into the personal experiences, perceptions, and self-assessed health outcomes of survivors, complementing the clinical data typically used in long-term follow-up studies. Our study features an extended follow-up period and investigates survivorship into adulthood. While much of the existing literature focuses on shorter-term follow-up, our long-term perspective is critical for understanding the enduring effects of ALL and its treatment, providing a comprehensive view of survivorship well beyond the immediate posttreatment years. Self-reported health outcomes could, together with risks due to prior cancer treatment, be used as predictors for the risk of chronic health conditions. As the number of adult survivors of childhood cancer is on the rise, there is an increase in the demand for improvement of the efficacy of follow-up centers. These characteristics allow us to use the health outcomes identified in this study to inform the development of follow-up services specifically tailored to the needs of long-term survivors of ALL. In doing so, we aim to contribute to the design of proactive need-based health care services that align with the preferences of long-term survivors, leveraging the advancements in long-term follow-up services developed in Sweden in recent years.

Our findings underscore the potential of long-term follow-up services that extend beyond targeting specific health conditions to encompass the broader spectrum of vitality, sleep quality, fatigue, and psychosocial well-being. Such comprehensive approaches could not only enhance well-being but also bolster the capacity of survivors to cope with chronic health conditions and reduce the likelihood of more severe health complications in the future. While many adult survivors of childhood ALL may not exhibit signs of chronic health conditions in their young adult years and regular clinic visits could potentially be more burdensome than beneficial, a tailored health screening approach introduced in a feasible digital format could serve as an excellent alternative. Such screening could effectively identify survivors who require in-person visits to the long-term follow-up clinic or offer health-promoting guidance to prevent the onset of chronic health conditions. Importantly, the value of digital interventions targeting prevention and promotion in relation to vitality, sleep quality, fatigue, and psychosocial well-being goes beyond these specific health conditions. They can potentially contribute to a holistic strategy to enhance the overall well-being of survivors and strengthen their capacity to manage chronic health conditions effectively. Such efforts to reduce the likelihood of more severe health complications in the future are of paramount importance for this group.

### Strengths and Limitations

Our study has several strengths, including its extended follow-up period, which distinguishes it from many other studies that have primarily concentrated on evaluating health outcomes in younger survivors [62]. This long-term focus provides valuable insights into the vulnerability of adult survivors of childhood ALL to negative health outcomes during adulthood. Additionally, comparisons with siblings who shared similar backgrounds, social environments, and childhood exposures enrich the depth of our findings [63]. Furthermore, the use of validated questionnaires bolsters the reliability of our results.

However, there are certain limitations inherent to our study that necessitate consideration. It is important to acknowledge that the psychosocial well-being of the siblings may have been directly or indirectly affected by the distress associated with the treatment and rehabilitation of the patients with ALL. Consequently, the use of siblings as a comparison group might potentially underestimate the impact on psychosocial problems when compared to the general population.

The response rate from adult survivors of childhood ALL was relatively low overall. Although nonresponders did not exhibit substantial differences from responders, it is crucial to recognize that a significant proportion of the population was missing from our analysis. Additionally, the smaller number of siblings compared to adult survivors of childhood ALL limits the extent to which we can compare these groups. Finally, our study was constrained by the limited treatment data available in the Swedish childhood leukemia registry, which precludes further in-depth analyses of the associations between ALL treatment and reported symptoms.

### Conclusions

Our population-based cross-sectional study provides valuable insights into the self-reported health outcomes among adult survivors of childhood ALL. Our findings underscore the potential of long-term follow-up services to extend beyond targeting specific health conditions, encompassing vitality, sleep quality, fatigue, and psychosocial well-being. Such comprehensive approaches could enhance well-being, bolster the capacity of survivors to manage chronic conditions, and reduce the likelihood of severe health complications. For many survivors, tailored digital follow-up of health issues and everyday functioning could be an excellent alternative to physical visits to a clinic, effectively identifying those who need in-person care or providing preventive health guidance. Such a holistic approach could promote participation in long-term follow-up and could improve the overall well-being and long-term health outcomes of childhood ALL survivors through tailored offers of services and interventions.

## Appendix

Multimedia Appendix 1 Questionnaire to survivors of acute lymphoblastic leukemia.

Multimedia Appendix 2 Questionnaire to siblings of survivors of acute lymphoblastic leukemia.

## Abbreviations

ALL: acute lymphoblastic leukemia
CVD: cardiovascular disease
CWP: chronic widespread pain
HRQoL: health-related quality of life
HSCT: hematopoietic stem cell transplantation

## Data Availability

The data that support the findings of this study are available upon reasonable request.
