# Supplementary material for: Charting Health Challenges for Digital Preventive Interventions Among Adult Survivors of Childhood Acute Lymphoblastic Leukemia: National Long-Term Follow-Up Survey of Self-Rated Health Outcomes
Source: JMIR Form Res. 2024 Aug 12;8:e54819. doi: 10.2196/54819 (PMC11347897; doi:10.2196/54819)
Supplement: Multimedia Appendix 1 [file formative_v8i1e54819_app1.pdf]

Machine Translated by Google

HÖGSKOLAN
I HALMSTAD

Health after treatment for childhood leukemia

1. Initial page

* 1. Before you have the opportunity to start answering the questions, you must first enter your personal code here, which you received in the information letter about the study.

Machine Translated by Google

HÖGSKOLAN I HALMSTAD

Health after treatment for childhood leukemia

2. Informed consent to participate in the research study **I have read the information about the research study Health-related quality of life and buffering factors in adults treated for pediatric acute lymphoblastic leukemia (ALL) and their siblings - a longitudinal study. I have received information about the purpose of the research study, which is to investigate how people who have been treated for leukemia as children and their siblings feel as adults, regarding both physical and psychosocial factors, as well as how they estimate their health-related quality of life.**

My participation means that I will answer a questionnaire on one occasion. This consent also means that I approve that information documented in the National Board of Health and Welfare's, the Social Insurance Agency's and Statistics Sweden's registers may be used in the research study. My answers to the questions and also information from various registers will be de-identified and reported only at group level.

I have read the information that participation in the research study is voluntary and that I can cancel my participation at any time without explanation without reason or with any consequences. If I wish to cancel my participation in the research study, I will notify the researchers.

* 2. Have you read the information letter and agree to participate in the research study?

- ◯ No, I do not agree to participate in the research study
- ◯ Yes, I agree to participate in the research study

If you agree to participate in the research study, write your initials here

[ ]

Machine Translated by Google

HÖGSKOLAN I HALMSTAD

Health after treatment for childhood leukemia

3. Instructions for completing this questionnaire **The questionnaire is based on self-assessment, which means that it is you who assess your own characteristics. You can therefore neither answer the questions right nor wrong, but the most important thing is that you leave the answers that you feel at the moment best describe how you feel or think. Also, be sure to answer all the questions.**

Since the covid-19 pandemic has affected society in recent months, the question is asked after each section in the survey about how much of an impact the covid-19 pandemic has had on you and thus your answers in each section.

The questionnaire can take up to about 40 minutes to fill in. Choose an environment that is quiet, calm and without disturbing elements.

Machine Translated by Google

HÖGSKOLAN I HALMSTAD

Health after treatment for childhood leukemia

4. Family, education, work

In this paragraph you must describe a little about your family, your education and what you do or work with.

* 3. Were you born in Sweden?

- Yes
- No, in another Nordic country
- No, in another country in Europe
- No, in another country outside Europe

* 4. Where was your mother born?

- My mother was born in Sweden
- My mother was born in another Nordic country
- My mother was born in another country in Europe
- My mother was born in another country outside Europe
- Other

* 5. Where was your father born?

- My father was born in Sweden
- My father was born in another Nordic country
- My father was born in another country in Europe
- My father was born in another country outside Europe
- Other

Machine Translated by Google

* 6. Do you have any siblings?

- No
- Yes. Enter how many siblings you have.

* 7. Do you have a life partner?

- Yes I'm married
- Yes, I have a partner
- Yes, I am in a relationship
- No, I'm single
- Other

* 8. Do you have children of your own?

- No
- Yes. Enter how many children you have.

* 9. What is your highest completed education?

- Elementary school
- Secondary school or vocational school
- College/university - less than 3 years
- College/University - 3 years or more

* 10. What is your main occupation today?

- I study
- I am working
- I am unemployed
- I am on maternity leave
- I am on sick leave
- I am voluntarily on leave
- Other

11. If you work, what do you work as? (enter occupation)

12. If you are on sick leave, enter the extent of sick leave in percentage

* 13. If we assume that your work ability, when it was at its best, is valued with 10 points. Which score would you then give your current working ability?
Mark the appropriate number (0 means that you cannot work at all now and 10 that your ability to work is at its best right now).

0	1	2	3	4	5	6	7	8	9	10
○	○	○	○	○	○	○	○	○	○	○

* 14. What is your monthly income? (gross, i.e. before tax)

- ○ Less than SEK 10,000
- ○ SEK 10,000 - SEK 19,999
- ○ SEK 20,000 - SEK 29,999
- ○ SEK 30,000 - SEK 39,999
- ○ SEK 40,000 - SEK 49,999
- ○ SEK 50,000 – SEK 59,999
- ○ SEK 60,000 or more

* 15. Have you sought care at any time during the past 12 months?

Enter type of care and number of visits

	0 times	1-5 times	6-10 times	11-20 times	21-40 times	>40 times
Follow-up reception/Late effects reception	○	○	○	○	○	○
Oncologist	○	○	○	○	○	○
Other specialist doctor	○	○	○	○	○	○
Doctor at health center	○	○	○	○	○	○
District nurse	○	○	○	○	○	○
Physiotherapist	○	○	○	○	○	○
Chiropractor/Naprapat	○	○	○	○	○	○

Other, namely

* 16. Do you have any kind of contact with the clinic where you were treated as a child?

- Yes
- No
- Do not know

* 17. Do you go for regular check-ups due to your illness as a child?

- Yes, to Follow-up Reception/Late Effects Reception
- Yes, to another clinic within adult healthcare
- No
- Do not know

* 18. Are you satisfied with the contact you have with the healthcare system related to your illness as a child?

- I am satisfied with the contact I have
- I'm happy with no contact
- I am dissatisfied with the contact I have
- I am unhappy with no contact
- Do not know

* 19. To what extent has the covid-19 pandemic affected your answers to the questions about family, education and work?

0 = Not affected in some degree at all	1	2	3	4	5	6	7	8	9	10 = Affected in very High grade

Machine Translated by Google

HÖGSKOLAN
I HALMSTAD

Health after treatment for childhood leukemia

5. Physical activity

Choose for each question the option that best describes your physical activity during the last month.

* 20. In a typical week, how much time do you spend on **physical exercise** that makes you tired, such as running, gymnastics or ball sports?

- 0 minutes/No time
- Less than 30 minutes
- 30-60 minutes (0.5-1 hour)
- 60-90 minutes (1-1.5 hours)
- 90-120 minutes (1.5-2 hours)
- More than 120 minutes (2 hours)

* 21. How much time do you spend in a typical week on **everyday exercise,** for example walking, cycling or gardening?
Add up all time (include time consisting of at least 10 minutes of continuous exercise)

- 0 minutes/No time
- Less than 30 minutes
- 30-60 minutes (0.5-1 hour)
- 60-90 minutes (1-1.5 hours)
- 90-150 minutes (1.5-2.5 hours)
- 150-300 minutes (2.5-5 hours)
- More than 300 minutes (5 hours)

* 22. How much do you **sit** during a normal day, excluding sleep?

- Pretty much all day
- 13-15 hours
- 10-12 hours
- 7-9 hours
- 4-6 hours
- 1-3 hours
- Never

* 23. What is your height and weight?

Length in centimeters (cm) __________

Weight in kilograms (kg) __________

* 24. To what extent has the covid-19 pandemic affected your answers to the questions about physical activity?

0 = Not affected in some degree at all	1	2	3	4	5	6	7	8	9	10 = Affected in very High grade
○	○	○	○	○	○	○	○	○	○	○

Machine Translated by Google

HÖGSKOLAN I HALMSTAD

Health after treatment for childhood leukemia

6. Sleep

Choose for each question the option that best describes your sleep during the last month.

* 25. How would you describe the quality of your sleep?

- Good
- Enough
- Bad

* 26. How many hours do you sleep a night?

- Less than 5 hours
- 5-7 hours
- 7-9 hours
- More than 9 hours

* 27. Do you wake up at night?

- Yes
- No

Machine Translated by Google

HÖGSKOLAN I HALMSTAD

Health after treatment for childhood leukemia

7.

* 28. If you wake up at night, what is the most common reason?

- [ ] Toilet visit
- [ ] Nightmare
- [ ] Hunger
- [ ] Waking up too early
- [ ] Fear or worry
- [ ] Pain
- [ ] Other

* 29. To what extent has the covid-19 pandemic affected your answers to the questions about sleep?

0 = Not affected in some degree at all	1	2	3	4	5	6	7	8	9	10 = Affected in very High grade
○	○	○	○	○	○	○	○	○	○	○

Machine Translated by Google

HÖGSKOLAN
I HALMSTAD

Health after treatment for childhood leukemia

8. Alcohol and tobacco

Choose for each question the option that best describes your consumption of alcohol and tobacco in the past months.

* 30. How often do you drink alcohol?

- ○ Never
- ○ Once a month or less often
- ○ 2-4 times a month
- ○ 2-3 times a week
- ○ 4 times a week or more

HÖGSKOLAN I HALMSTAD

Health after treatment for childhood leukemia

9.

* 31. How many "glasses" (see example below) do you drink on a typical day when you drink alcohol?

- 1-2 glasses
- 3-4 glasses
- 5-6 glasses
- 7-9 glasses
- 10 or more glasses

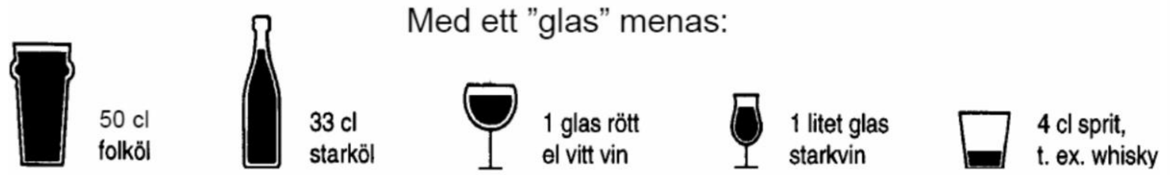

* 32. How often do you use tobacco?

- Never
- Some day of the month
- Some day a week
- Several days a week
- Every day

HÖGSKOLAN I HALMSTAD

Health after treatment for childhood leukemia

10.

* 33. How many cigarettes do you consume on a typical day when you use tobacco?

- 0 pcs
- 1-2 pcs
- About 5 pcs
- About 10 pcs
- One package or more

* 34. How many portions of snuff do you consume on a typical day when you use tobacco?

- 0 pcs
- 1-2 pcs
- About 5 pcs
- About 10 pcs
- A can or more

* 35. To what extent has the covid-19 pandemic affected your answers to the questions about alcohol and tobacco?

0 = Not affected in some degree at all	1	2	3	4	5	6	7	8	9	10 = Affected in very High grade
○	○	○	○	○	○	○	○	○	○	○

Machine Translated by Google

HÖGSKOLAN I HALMSTAD

Health after treatment for childhood leukemia

11. Health

Answer the questions by marking the word you think best describes you.

If you are unsure, still mark the word that feels most correct.

* 36. In general, would you say your health is?

- Excellent
- Very good
- Good
- Fairly good
- Bad

* 37. Compared to a year ago, how would you rate your general state of health now? __

- Much better now than a year ago
- Slightly better now than a year ago
- About the same
- Slightly worse now than a year ago
- Much worse now than a year ago

* 38. The following questions are about activities that you can imagine doing during a normal day. Are you currently limited in these activities due to your health condition? If so, how much?

	Yes, very limited	Yes, a bit limited	No, not limited at all
**Strenuous activities,** such as running, lifting heavy objects, participating in strenuous sports	○	○	○
**Moderately strenuous activities,** such as moving a table, vacuuming, forest walks or gardening	○	○	○
Lift or carry grocery bags	○	○	○
Go up **several** stairs	○	○	○
Go up **a** flight of stairs	○	○	○
Bend over or get down on your knees	○	○	○
Go **more than two kilometers**	○	○	○
Walk **a few hundred meters**	○	○	○
Walk **a hundred meters**	○	○	○
Wash or dress	○	○	○

* 39. During the past four weeks, have you had any of the following problems in your work or with other regular daily activities as a result of your physical health condition?

	Yes	No
Cut down the **time** you normally devoted to work or other activities	○	○
**Accomplished less** than you would have liked	○	○
Been prevented from performing **certain** tasks or other activities	○	○
Had **difficulties** to perform your work or other activities (eg by requiring extra effort)	○	○

* 40. During the past four weeks, have you had any of the following problems in your work or with other regular daily activities as a result of emotional problems (such as low mood or anxiety)?

	Yes	No
Cut down the **time** you normally devoted to work or other activities	○	○
**Accomplished less** than you would have liked	○	○
Not done work or other activities so **carefully** as usual	○	○

* 41. During the past four weeks, to what extent has your physical health condition or emotional problems interfered with your usual interactions with relatives, friends, neighbors or others?

- Not at all
- A little
- Moderately
- Very
- Very much

* 42. How much ache or pain have you had in the past four weeks?

- No
- Very easy
- Easy
- Moderate
- Difficult
- Very difficult

* 43. During the past four weeks, how much has ache or pain interfered with your normal work (both include work outside the home and household chores)?

- Not at all
- A little
- Moderately
- Very
- Very much

* 44. The questions here are about how you feel and how you have been in the last four weeks. For each question, enter the answer option that best describes how you felt. How much of the time in the last four weeks…

	Continuously	Most of the the time	A lot of the time	Some of the time	A little of the time	No part of the time
Have you felt really energetic and strong?						
Have you been feeling very nervous?						
Have you felt that way? downcast that nothing could cheer you up?						
Have you felt calm and harmonious?						
Have you been full of energy?						
Have you been feeling gloomy and sad?						
Have you been feeling exhausted?						
Have you felt joyful and happy?						
Have you been feeling tired?						

* 45. During the past four weeks, how much of the time did your physical health condition or emotional problems interfere with your ability to socialize (eg see family, friends, etc.)?

- Continuously
- Most of the time
- Some of the time
- A little of the time
- None of the time

* 46. Choose the answer option that best describes the extent to which each of the following statements is TRUE or DOESN'T suit you.

	Fits exactly	Quite true Good	Uncertain	Incorrect especially good	Not correct at all
I seem to have it a little easier to get sicker than others people	○	○	○	○	○
I'm as healthy as anyone any of them i feel	○	○	○	○	○
I believe my health will get worse	○	○	○	○	○
My health is excellent	○	○	○	○	○

* 47. To what extent has the covid-19 pandemic affected your answers to the questions about health?

0 = Not affected in some degree at all	1	2	3	4	5	6	7	8	9	10 = Affected in very High grade
○	○	○	○	○	○	○	○	○	○	○

Machine Translated by Google

HÖGSKOLAN
I HALMSTAD

Health after treatment for childhood leukemia

12. Self-confidence

* 48. Indicate for each statement how well they describe you as a person.

	Totally distance myself from	Partially dissociates from	Partially agree with	Totally agree with
I always manage to solve hard problems if i just making an effort enough.	◯	◯	◯	◯
Even if someone oppose me I find still ways to reach mine goal.	◯	◯	◯	◯
I have no difficulties to hold on to mine objectives and realize my goals.	◯	◯	◯	◯
In unexpected situations I always know how to do it act.	◯	◯	◯	◯
Even surprising situations I think I can cope well.	◯	◯	◯	◯
Thanks to my own I feel capable calm even when I am asked in the face of difficulties.	◯	◯	◯	◯
Whatever happens I always manage.	◯	◯	◯	◯
Whatever problem I faced I can find a solution.	◯	◯	◯	◯
If I am faced with new challenges I know how I will deal with them.	◯	◯	◯	◯
When problems arise can I usually manage them by their own power.	◯	◯	◯	◯

* 49. To what extent has the covid-19 pandemic affected your answers to the questions about self-confidence?

0 = Not affected in some degree at all	1	2	3	4	5	6	7	8	9	10 = Affected in very High grade
◯	◯	◯	◯	◯	◯	◯	◯	◯	◯	◯

HÖGSKOLAN
I HALMSTAD

Health after treatment for childhood leukemia

13. Social support

* 50. How many people do you know and have contact with, who have the same interests as you?

- No
- 1-2
- 3-5
- 6-10
- 11-15
- More than 15

* 51. How many people, that you know, do you meet or talk to during a typical week?
(Don't count on people you meet casually and who you will hardly see again)

- No
- 1-2
- 3-5
- 6-10
- 11-15
- More than 15

* 52. How many friends do you have who can come to your house at any time and feel at home?
(They wouldn't care if it was dirty or if you were eating. Close relatives don't count)

- No
- 1-2
- 3-5
- 6-10
- 11-15
- More than 15

* 53. How many people are there, in your family and among your friends, with whom you can speak openly without having to think twice?

- No
- 1-2
- 3-5
- 6-10
- 11-15
- More than 15

* 54. Do you have someone you can share your innermost feelings with and confide in?

- Yes
- No

* 55. Does it happen that someone holds you for comfort or support?

- Yes
- No

* 56. Is there a particular person who you think feels very close to you?

- Yes
- Am not sure
- No

* 57. Do you have a special person with whom you can share your feelings when you feel happy? Someone who would feel happy just because you are?

- Yes
- No

Machine Translated by Google

* 58. Is there a particular person that you feel you can really get support from?

- Yes
- Yes, but I don't need it
- No

* 59. Do you think that those at home or others really appreciate what you do for them?

- Yes
- Not enough
- Not at all

* 60. How many people in your environment are there that you can easily ask for things?
(E.g. people you know so well that you can borrow tools or kitchen utensils)

- No
- 1-2
- 3-5
- 6-10
- 11-15
- More than 15

* 61. Apart from those at home, is there anyone you can turn to if you are in difficulty?
(Someone whom you can easily meet and whom you trust and can get real help from)

- Yes
- No

Machine Translated by Google

HÖGSKOLAN
I HALMSTAD

Health after treatment for childhood leukemia

14.

* 62. How many such people do you have access to?

- No
- 1-2
- 3-5
- 6-10
- 11-15
- More than 15

* 63. When I have planned something, I also carry it out.

1 = No, true not	2	3	4	5	6	7 = Yes, correct completely
○	○	○	○	○	○	○

* 64. I usually manage in one way or another.

1 = No true not	2	3	4	5	6	7 = Yes, agree completely
○	○	○	○	○	○	○

* 65. I trust myself more than anyone else

1= No, agree not	2	3	4	5	6	7 = Yes, agree completely
○	○	○	○	○	○	○

* 66. Being interested in different things is important to me.

1 = No agree not	2	3	4	5	6	7 = Yes, agree completely
○	○	○	○	○	○	○

* 67. I can be alone if I have to.

1 = No, does not agree	2	3	4	5	6	7 = Yes, agree completely
○	○	○	○	○	○	○

* 68. I feel proud of having achieved something in my life.

1 = No, does not agree	2	3	4	5	6	7 = Yes, agree completely
○	○	○	○	○	○	○

* 69. Usually I take things as they come.

1 = No, true not	2	3	4	5	6	7 = Yes, correct completely
○	○	○	○	○	○	○

* 70. I am friends with myself.

1 = No, does not agree	2	3	4	5	6	7 = Yes, agree completely
○	○	○	○	○	○	○

* 71. I feel that I can keep "many balls in the air" at the same time.

1 = No, does not agree	2	3	4	5	6	7 = Yes, agree completely
○	○	○	○	○	○	○

* 72. I am determined.

1 = No, does not agree	2	3	4	5	6	7 = Yes, agree completely
○	○	○	○	○	○	○

* 73. I rarely ponder the meaning of everything.

1 = No, does not agree	2	3	4	5	6	7 = Yes, agree completely
○	○	○	○	○	○	○

* 74. I take one day at a time.

1 = No, does not agree	2	3	4	5	6	7 = Yes, agree not
○	○	○	○	○	○	○

* 75. I can cope with difficult times because I have experienced difficulties before.

1 = No, does not agree	2	3	4	5	6	7 = Yes, agree completely
○	○	○	○	○	○	○

* 76. I have self-discipline.

1 = No, does not agree	2	3	4	5	6	7 = Yes, agree completely
○	○	○	○	○	○	○

* 77. I am curious about what is happening and happening.

1 = No, does not agree	2	3	4	5	6	7 = Yes, agree completely
○	○	○	○	○	○	○

* 78. I usually find something to be happy about.

1 = No, does not agree	2	3	4	5	6	7 = Yes, agree completely
○	○	○	○	○	○	○

* 79. My belief in myself helps me through difficulties.

1 = No, does not agree	2	3	4	5	6	7 = Yes, agree completely
○	○	○	○	○	○	○

* 80. In an emergency, I am a person that people can usually trust.

1 = No, does not agree	2	3	4	5	6	7 = Yes, agree completely
○	○	○	○	○	○	○

* 81. Usually I can look at a situation in many different ways.

1 = No, does not agree	2	3	4	5	6	7 = Yes, agree completely
○	○	○	○	○	○	○

* 82. Sometimes I force myself to do things whether I want to or not.

1 = No, does not agree	2	3	4	5	6	7 = Yes, agree completely
○	○	○	○	○	○	○

* 83. My life feels meaningful.

1 = No, does not agree	2	3	4	5	6	7 = Yes, agree completely
○	○	○	○	○	○	○

* 84. I don't dwell on things I can't do anything about.

1 = No, does not agree	2	3	4	5	6	7 = Yes, agree completely
○	○	○	○	○	○	○

* 85. When I find myself in a difficult situation, I usually find a solution.

1 = No, does not agree	2	3	4	5	6	7 = Yes, agree completely
○	○	○	○	○	○	○

* 86. I have enough energy to do what I have to.

1 = No, does not agree	2	3	4	5	6	7 = Yes, agree completely
○	○	○	○	○	○	○

* 87. I can accept not being liked by everyone.

1 = No, does not agree	2	3	4	5	6	7 = Yes, agree completely
○	○	○	○	○	○	○

* 88. To what extent has the covid-19 pandemic affected your answers to the questions about social support?

0 = Not affected in some degree at all	1	2	3	4	5	6	7	8	9	10 = Affected in very High grade
○	○	○	○	○	○	○	○	○	○	○

HÖGSKOLAN
I HALMSTAD

Health after treatment for childhood leukemia

15. Sense of context

Below are questions and statements that concern different areas of life. Please choose one option that best matches your feeling. If in doubt, choose the option that feels right most true.

* 89. Do you have the feeling that you don't really care about what is happening around you?

1 = Very rarely or never	2	3	4	5	6	7 = Very often
○	○	○	○	○	○	○

* 90. Has it ever happened that you were surprised by the behavior of people you thought you knew well?

1 = Never have happened	2	3	4	5	6	7 = Has often happened
○	○	○	○	○	○	○

* 91. Has it happened that people you trusted have disappointed you?

1 = Never have happened	2	3	4	5	6	7 = Has often happened
○	○	○	○	○	○	○

* 92. So far your life has:

1 = Completely missing goal and meaning	2	3	4	5	6	7 = Throughout had goals and sentence
○	○	○	○	○	○	○

Machine Translated by Google

* 93. Do you feel unfairly treated?

1 = Very often	2	3	4	5	6	7 = A lot rarely/never
○	○	○	○	○	○	○

* 94. Do you have the feeling that you are in an unfamiliar situation (e.g. in case of illness) and do not know what to do do?

1 = Very often	2	3	4	5	6	7 = A lot rarely/never
○	○	○	○	○	○	○

* 95. Are your daily chores a source of:

1 = Joy and depth satisfaction	2	3	4	5	6	7 = Pain and lead
○	○	○	○	○	○	○

* 96. Do you have a lot of conflicting feelings and thoughts?

1 = Very often	2	3	4	5	6	7 = A lot rarely/never
○	○	○	○	○	○	○

* 97. Does it happen that you have feelings inside you that you would rather not feel?

1 = Very often	2	3	4	5	6	7 = A lot rarely/never
○	○	○	○	○	○	○

* 98. Even a person with strong self-esteem can sometimes feel like an "unlucky bird". How often have you felt that so?

1 = Never	2	3	4	5	6	7 = Very often
○	○	○	○	○	○	○

* 99. When something has happened, you have usually found that:

1 = You over- or undervalued its meaning	2	3	4	5	6	7 = You saw the thing in its right proportion
○	○	○	○	○	○	○

* 100. How often do you feel that there is no meaning to the things you do in your daily life?

1 = Very often	2	3	4	5	6	7 = A lot rarely/never
○	○	○	○	○	○	○

* 101. How often do you have feelings that you are not sure you can control?

1 = Very often	2	3	4	5	6	7 = A lot rarely/never
○	○	○	○	○	○	○

* 102. To what extent has the covid-19 pandemic affected your answers to the questions about sense of context?

0 = Not affected in some degree at all	1	2	3	4	5	6	7	8	9	10 = Affected in very High grade
○	○	○	○	○	○	○	○	○	○	○

Health after treatment for childhood leukemia

16. Mental health

* 103. Read each statement and mark the option that best indicates how much the statement resonated with you in the past week.

There are no right or wrong answers. Don't spend too much time on any statement.

	Didn't suit me	Agreed with me to some extent or some of the time	Agreed with me to a great extent or a large part of the time	Fit me very well or for the most part
I had a hard time winding down down.	○	○	○	○
I was aware of dry mouth.	○	○	○	○
I couldn't seem to experience any positive feeling at all.	○	○	○	○
I experienced breathing difficulties (eg, excessive rapid breathing, shortness of breath in the absence of physical exertion.	○	○	○	○
I had difficulty taking the initiative to do things.	○	○	○	○
I tended to overreact to situations.	○	○	○	○
I experienced tremors (eg in the hands).	○	○	○	○
I felt like I used a lot energy to nervousness.	○	○	○	○

	Didn't suit me	Agreed with me to some extent or some of the time	Agreed with me to a great extent or a large part of the time	Fit me very well or for the most part
I was worried about situations in which I might panic and cut myself off.	○	○	○	○
I felt like I had nothing to look forward to against.	○	○	○	○
I found myself getting upset.	○	○	○	○
I had a hard time relaxing.	○	○	○	○
I felt down and depressed.	○	○	○	○
I was intolerant of anything that kept me from moving forward with what I was doing.	○	○	○	○
I felt like I was close to panicking.	○	○	○	○
I was unable to get excited about anything.	○	○	○	○
I felt like I wasn't worth much person	○	○	○	○
I felt that I was quite easily supported.	○	○	○	○
I was aware of the work of my heart in the absence of physical exertion (eg feeling that the heart skips a beat).	○	○	○	○
I felt scared for no good reason.	○	○	○	○
I felt that life was meaningless.	○	○	○	○

* 104. To what extent has the covid-19 pandemic affected your answers to the questions about mental health?

0 = Not affected to some degree at all	1	2	3	4	5	6	7	8	9	10 = Affected in very high degree
○	○	○	○	○	○	○	○	○	○	○

Machine Translated by Google

Machine Translated by Google

HÖGSKOLAN I HALMSTAD

Health after treatment for childhood leukemia

17. Physical health

* 105. Have you ever had a heart attack?

No

Yes

* 106. Have you ever been treated for heart failure?
(You may have experienced a feeling of not getting air (shortness of breath) and your doctor may have said that you have fluid in your lungs or that your heart is not pumping well enough.)

No

Yes

* 107. Have you ever had surgery to clear or bypass arteries in your legs?

No

Yes

* 108. Have you ever had any of the following; a stroke, damage to the brain's blood vessels (cerebrovascular accident), blood clot or bleeding in the brain, or a temporary disruption of blood flow to part of the brain (transient ischemic attack, TIA)?

No

Yes

* 109. Do you have difficulty moving your arms or legs as a result of a stroke or damage to the brain's blood vessels (cerebrovascular accident)?

No

Yes

* 110. Do you have asthma? If yes, do you take medication for your asthma?

- No
- Yes, but only in case of difficulty breathing
- Yes, I take medicine regularly, even when I don't have difficulty breathing

* 111. Do you have emphysema, chronic bronchitis, or chronic obstructive pulmonary disease (COPD)?

If yes, do you take medicine for your lung disease?

- No
- Yes, but only in case of difficulty breathing
- Yes, I take medicine regularly, even when I don't have difficulty breathing

* 112. Do you have a stomach ulcer?

If yes, was this condition diagnosed by endoscopy or by x-ray?

Endoscopy - the inside of the body is studied via the esophagus with an instrument equipped with a camera

X-ray - you swallow contrast medium and then x-rays are taken of your stomach

- No
- Yes, diagnosed by endoscopy
- Yes, diagnosed by x-ray

* 113. Do you have diabetes (high blood sugar)?

- No
- Yes, treated by adjusting my diet
- Yes, treated with medications taken by mouth
- Yes, treated with insulin injections

Machine Translated by Google

HÖGSKOLAN I HALMSTAD

Health after treatment for childhood leukemia

18.

* 114. If you have diabetes, has it caused any of the following problems?

	Yes	No
Problems with the kidneys	◯	◯
Problems with the eyes, treated by one ophthalmologist	◯	◯

* 115. Have you ever had the following problems with your kidneys?

	Yes	No
Impaired kidney function (blood tests show high levels of creatinine)	◯	◯
Has been treated with dialysis (hemodialysis or peritoneal dialysis)	◯	◯
Has undergone a kidney transplant	◯	◯

* 116. Do you have rheumatism (rheumatoid arthritis)?
If yes, do you take medication regularly?

◯ No

◯ Yes, but without regular medication

◯ Yes, I take medication regularly

* 117. Do you have any of the following diseases?

	Yes	No
Systemic lupus erythematosus (SLE)	○	○
Polymalgia rheumatica (inflammatory muscular rheumatism)	○	○

* 118. Do you currently have one or more of the following diseases or problems?

	Yes	No
Tension headache	○	○
Migraine	○	○
Alzheimer's disease or other form of dementia disease	○	○
Liver cirrhosis (cirrhosis) or severe liver damage	○	○
Leukemia	○	○
Lymphoma	○	○
Other cancer	○	○
If you have cancer, has it spread (metastasized) to other parts of the body?	○	○
AIDS	○	○

Machine Translated by Google

HÖGSKOLAN
I HALMSTAD

Health after treatment for childhood leukemia

19. Aches and pains **The**

questions refer to aches and pains in joints and muscles/soft tissues that are persistent or regularly recurring. Mark the one that suits you best.

* 119. During the past 12 months, have you had aches or pains that lasted more than 3 months?

- Yes
- No
- Do not know

Machine Translated by Google

HÖGSKOLAN I HALMSTAD

Health after treatment for childhood leukemia

20.

* 120. Do you still have aches or pains?

- ○ Yes
- ○ No

* 121. Mark one or more of the boxes below, in all the places on the body where you experienced aches or pain for more than 3 months in the last 12 months!

Note that the small letters refer to specific areas of the body that are delineated in the figure below.

- ☐ a. The front of the chest
- ☐ b. Neck
- ☐ c. Weave shoulder/upper arm
- ☐ d. High shoulder/upper arm
- ☐ e. Arm elbow/forearm
- ☐ f. High elbow/forearm
- ☐ g. Thoracic spine
- ☐ h. Lumbar/lower back
- ☐ i. Hand/wrist
- ☐ j. Hay hand/wrist
- ☐ k. Vä ham
- ☐ l. Hay ham
- ☐ m. So hip/thigh
- ☐ n. High hip/thigh
- ☐ o. Woe knee
- ☐ p. Hay knee
- ☐ q. Lower leg/foot
- ☐ r. Lower leg/foot

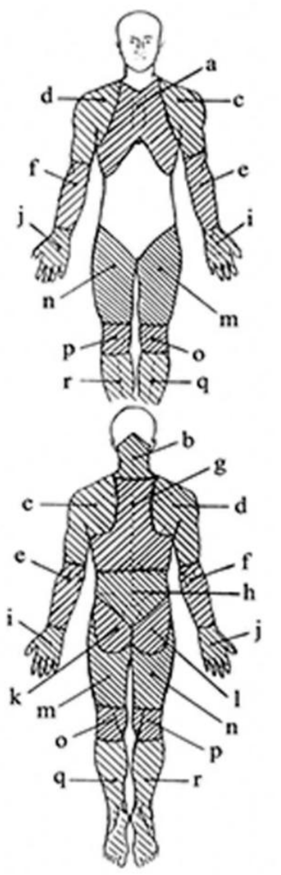

* 122. How much pain have you had in the past week?

0 = None pain	1	2	3	4	5	6	7	8	9	10 = Worst imaginable pain
○	○	○	○	○	○	○	○	○	○	○

* 123. To what extent has the covid-19 pandemic affected your answers to the questions about aches and pains?

0 = Not affected in some degree at all	1	2	3	4	5	6	7	8	9	10 = Affected in very High grade
○	○	○	○	○	○	○	○	○	○	○

Machine Translated by Google

HÖGSKOLAN I HALMSTAD

Health after treatment for childhood leukemia

21. Fatigue

Through the following statements, we want to get an idea of how you have felt in recent days.

* 124. I feel fit

1 = Yes, that's right	2	3	4	5 = No, that is not true
○	○	○	○	○

* 125. Physically, I feel able to do very little

1 = Yes, that's right	2	3	4	5 = No, that is not true
○	○	○	○	○

* 126. I feel very active

1 = Yes, that's right	2	3	4	5 = No, that is not true
○	○	○	○	○

* 127. I feel like doing a lot of nice things

1 = Yes, that's right	2	3	4	5 = No, that is not true
○	○	○	○	○

* 128. I feel tired

1 = Yes, that's right	2	3	4	5 = No, that is not true
○	○	○	○	○

* 129. I think I can get a lot done in one day

1 = Yes, that's right	2	3	4	5 = No, that is not true
○	○	○	○	○

Machine Translated by Google

* 130. When I do something, I can concentrate on it

1 = Yes, that's right	2	3	4	5 = No, that is not true
○	○	○	○	○

* 131. Physically, I can handle a lot

1 = Yes, that's right	2	3	4	5 = No, that is not true
○	○	○	○	○

* 132. I dread having to do something

1 = Yes, that's right	2	3	4	5 = No, that is not true
○	○	○	○	○

* 133. I get very little done in a day

1 = Yes, that's right	2	3	4	5 = No, that is not true
○	○	○	○	○

* 134. I find it easy to concentrate

1 = Yes, that's right	2	3	4	5 = No, that is not true
○	○	○	○	○

* 135. I am rested

1 = Yes, that's right	2	3	4	5 = No, that is not true
○	○	○	○	○

* 136. I use a lot of effort to concentrate on things

1 = Yes, that's right	2	3	4	5 = No, that is not true
○	○	○	○	○

* 137. Physically, I feel in bad shape

1 = Yes, that's right	2	3	4	5 = No, that is not true
○	○	○	○	○

* 138. I have lots of plans

1 = Yes, that's right	2	3	4	5 = No, that is not true
○	○	○	○	○

Machine Translated by Google

* 139. I get tired easily

1 = Yes, that's right	2	3	4	5 = No, that is not true
○	○	○	○	○

* 140. I don't get much done

1 = Yes, that's right	2	3	4	5 = No, that is not true
○	○	○	○	○

* 141. I have no desire to do anything

1 = Yes, that's right	2	3	4	5 = No, that is not true
○	○	○	○	○

* 142. My thoughts wander easily

1 = Yes, that's right	2	3	4	5 = No, that is not true
○	○	○	○	○

* 143. Physically, I feel in excellent shape

1 = Yes, that's right	2	3	4	5 = No, that is not true
○	○	○	○	○

* 144. To what extent has the covid-19 pandemic affected your answers to the questions about fatigue?

0 = Not affected in some degree at all	1	2	3	4	5	6	7	8	9	10 = Affected in very High grade
○	○	○	○	○	○	○	○	○	○	○

Machine Translated by Google

HÖGSKOLAN
I HALMSTAD

Health after treatment for childhood leukemia

22. Request to participate in a telephone interview

* 145. We intend to contact a number of people, who have answered the above questionnaire, for an in-depth telephone interview. The aim is to describe how adults who have been treated for leukemia in childhood perceive that their experiences from the treatment period have affected their current health, quality of life and lifestyle. Would you consider being contacted for a telephone interview?

- ○ No
- ○ Yes, please provide a phone number where we can reach you

Machine Translated by Google

HÖGSKOLAN I HALMSTAD

Health after treatment for childhood leukemia

23. Recruitment of siblings to control group **In this research study, the aim is to investigate how people who have been treated for leukemia as children feel as adults, regarding both physical and psychosocial factors, as well as how they estimate their health-related quality of life.**

Several of the factors measured in the research study are of a type that can be expected to be influenced by heredity and environment. Therefore, siblings of people treated for leukemia as children make the best control group for the research study.

If you have a sibling or siblings who you think would like to help us answer the same questions you answered in the questionnaire, please provide their name and address below. You can, of course, participate in the research study without providing information about siblings.

Siblings' participation in the research study is based on voluntariness and their possible answers will be protected by the same confidentiality as the answers you provided in your questionnaire. No one will be able to see if your sibling really answers any questions and it will never be possible to compare the answers from you and your siblings.

146. Sibling 1

Name	
Street address	
ZIP code	
Postal address	

147. Siblings 2

Name	
Street address	
ZIP code	
Postal address	

Machine Translated by Google

Machine Translated by Google

HÖGSKOLAN
I HALMSTAD

Health after treatment for childhood leukemia

24. Closing page **THANK YOU FOR YOUR PARTICIPATION.**

When the responses from all participants have been analyzed, you have the opportunity to follow the progress of the study and results/articles on the study's website.

Machine Translated by Google

HÖGSKOLAN I HALMSTAD

Health after treatment for childhood leukemia

25. Closing page in case of non-participation

Thank you for letting us know that you do not agree to participate in the research study.
